# The first genetic engineered system for ovothiol biosynthesis in diatoms reveals a mitochondrial localization for the sulfoxide synthase OvoA

**DOI:** 10.1098/rsob.220309

**Published:** 2023-02-01

**Authors:** Monia Teresa Russo, Anna Santin, Annalisa Zuccarotto, Serena Leone, Anna Palumbo, Maria Immacolata Ferrante, Immacolata Castellano

**Affiliations:** ^1^ Department of Research Infrastructures for Marine Biological Resources, Stazione Zoologica Anton Dohrn, Villa Comunale, Naples, Italy; ^2^ Department of Integrative Marine Ecology, Stazione Zoologica Anton Dohrn, Villa Comunale, Naples, Italy; ^3^ Department of Biology and Evolution of Marine Organisms, Stazione Zoologica Anton Dohrn, Villa Comunale, Naples, Italy; ^4^ Department of Molecular Medicine and Medical Biotechnology, University of Naples Federico II, 80131 Naples, Italy

**Keywords:** ovothiol, 5-thiohistidine, diatoms, marine antioxidants, *Phaeodactylum tricornutum*, sulfoxide synthase

## Abstract

Diatoms represent one of the most abundant groups of microalgae in the ocean and are responsible for approximately 20% of photosynthetically fixed CO_2_ on Earth. Due to their complex evolutionary history and ability to adapt to different environments, diatoms are endowed with striking molecular biodiversity and unique metabolic activities. Their high growth rate and the possibility to optimize their biomass make them very promising ‘biofactories’ for biotechnological applications. Among bioactive compounds, diatoms can produce ovothiols, histidine-derivatives, endowed with unique antioxidant and anti-inflammatory properties, and occurring in many marine invertebrates, bacteria and pathogenic protozoa. However, the functional role of ovothiols biosynthesis in organisms remains almost unexplored. In this work, we have characterized the thiol fraction of *Phaeodactylum tricornutum*, providing the first evidence of the presence of ovothiol B in pennate diatoms. We have used *P. tricornutum* to overexpress the 5-histidylcysteine sulfoxide synthase *ovoA*, the gene encoding the key enzyme involved in ovothiol biosynthesis and we have discovered that OvoA localizes in the mitochondria, a finding that uncovers new concepts in cellular redox biochemistry. We have also obtained engineered biolistic clones that can produce higher amount of ovothiol B compared to wild-type cells, suggesting a new strategy for the eco-sustainable production of these molecules.

## Graphical abstract

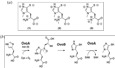

## 1. Introduction

Diatoms are photosynthetic unicellular eukaryotes representing one of the most abundant groups of microalgae in the ocean. They are responsible for approximately 20% of photosynthetically fixed CO_2_ on Earth, thus playing a key role in the marine ecosystem functioning. Diatoms are characterized by a complex evolutionary origin. Indeed, they contain genes related to those of both animals and plants, including the green algal lineage, as well as a rather high percentage of bacterial genes acquired through lateral gene transfer [1]. They are classified in two main groups differing in the symmetry of their silica wall: centric diatoms, evolutionary older, and the more recent pennate diatoms [2]. Among them, *Phaeodactylum tricornutum* represents one of the most studied pennate species with a fully sequenced genome [3]. During the last two decades, significant efforts have been devoted to the development of molecular tools to study diatom biology [4], as well as to establish microalgae as a significant, renewable and sustainable resource of biomass for feed, food, energy and other natural products [5–7]. Moreover, the development of tools for the genetic manipulation of diatoms is paving the way for a growing exploitation of these organisms in biotechnology. Indeed, *P. tricornutum* can be genetically transformed to produce high amounts of endogenous products or can be used as a cell factory to achieve the production of exogenous molecules of interest [8]. Exogenous genes can be inserted in *P. tricornutum* with different methods. Electroporation has found a limited use and it has never proven to be efficient, while the methods based on bacterial conjugation and on the biolistic bombardment of plasmid DNA on metal particles are widespread [9]. In addition, several papers have reported *P. tricornutum* as a model species for gene expression modulation: for example, this species is highly exploited for the modification of enzymes involved in lipid metabolism to increase lipid content [10–14].

**Figure 1.**
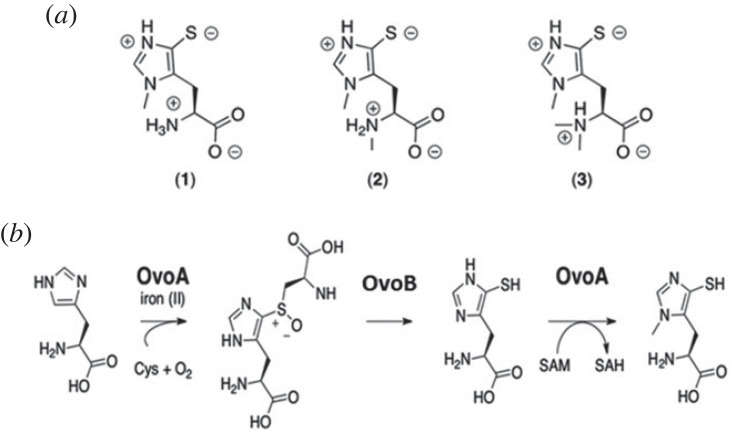
(*a*) Chemical structures of ovothiol A (1), B (2) and C (3). (*b*) Biosynthesis of ovothiol A.

Recent studies have highlighted that some species of microalgae produce, among other bioactive compounds, molecules belonging to the family of thiohistidines [15,16], sulfur-containing amino acids object of renewed attention thanks to their key properties in the removal of peroxides [17,18]. Thiohistidines comprise 2-thiohistidine and its tri-methylated derivative ergothioneine, which is mainly produced by some fungi and cyanobacteria [19], and 5-thiohistidine and its methylated forms, ovothiols, mainly present in marine invertebrates, bacteria and microalgae [20–22], both as free amino acids and as constituents of more complex molecular structures [20,21]. Thanks to the peculiar position of the thiol group on the imidazole ring of histidine, ovothiols are probably the most acidic natural thiols, a feature that confers them unique redox properties [17,18]. Ovothiols have been in fact proposed to play a key role in controlling the cellular redox balance, thanks to their ability to carry out redox exchange with glutathione (GSH) [23,24]. The three known forms of ovothiol, A, B and C (figure 1*a*) differ for the degree of methylation of the lateral chain of histidine [20]. Ovothiol A (1), unmethylated at the amino acidic amino group, was first isolated and characterized from the eggs of the sea urchin *Paracentrotus lividus* and was later detected also in other marine invertebrates [20,25,26], and recently in the green microalga *Euglena gracilis* [15]. Ovothiol B (2), mono-methylated at the lateral side chain, was first reported in the ovary of the scallop *Chlamys hastata* [27] and recently discovered in the diatom *Skeletonema marinoi* [16]. Ovothiol C (3), di-methylated at the amino acidic amino group, was isolated from other sea urchin species, like *Strongylocentrotus purpuratus* and *Sphaerechinus granularis* [20,25]*.* However, a more recent phylogenetic analysis suggests a widespread occurrence of these natural products in marine habitats, from bacteria to animals [28,29].

Ovothiols have also recently attracted the research community's interest for their therapeutic potential [21]. Ovothiol A is known to induce the arrest of cell proliferation through an autophagic mechanism in hepatocellular carcinoma cell lines [30]. In addition, it has been identified as a novel potent inhibitor of gamma-glutamyl-transpeptidase (GGT) activity in human cancer cells [31], able to ameliorate liver fibrosis [32]. Moreover, ovothiol A has been reported to exhibit anti-inflammatory activity in an *in vitro* model of endothelial dysfunction associated with diabetes [33], and in *ex-vivo* inflamed skin tissues [34]. In the past, a modified synthetic analogue of ovothiol was also shown to induce neuroprotective effect in mice affected by neuronal damage [35].

In nature, ovothiols are biosynthesized through three enzymatic reactions. The first reaction is catalysed by the 5-histidylcysteine sulfoxide synthase OvoA, a bifunctional enzyme, which, starting from cysteine and histidine, catalyses first the formation of the 5-histidyl-cysteine sulfoxide conjugate [36] and then the methylation of the imidazole ring of 5-thiohistidine, obtained after the cleavage of the sulfoxide intermediate by a pyridoxal phosphate-dependent lyase, OvoB [37] (figure 1*b*). The recent identification of the genes responsible for ovothiol biosynthesis in bacteria [36] has prompted pioneering functional studies on their role in other organisms [38]. Indeed, very little is known about the functional characterization of OvoA.

From the chemical point of view, the precursor of ovothiols, 5-thiohistidine, can be prepared by a feasible chemical synthesis according to the protocol described by Daunay *et al*. [39]. However, the synthesis of ovothiols, characterized by a methyl group at position π of the imidazole ring, is not feasible through this procedure, but similar compounds, the so-called iso-ovothiols, methylated at position *τ* of the imidazole ring, can be synthesized [40]. The antioxidant activity of iso-ovothiols has also been tested by measuring GSH peroxidase activity [41]. However, the biological activities of these ovothiols derivatives are currently unknown. On the other hand, up to now, ovothiols have been purified by sea urchin eggs in order to test their biological activities [30]. However, sea urchins are not an eco-sustainable source and cannot provide sufficient amounts of the natural compounds for extensive testing and transfer to clinical trials. In this context, microalgae, and diatoms in particular, may represent an optimal alternative natural source for ovothiol biosynthesis [16].

In the present manuscript, for the first time, we have cloned the *ovoA* gene of *P. tricornutum,* fused with a fluorescent tag, to overexpress the key enzyme involved in ovothiol biosynthesis, in order to follow its subcellular localization and to modulate its biosynthetic activity. By HPLC-MS analysis, we have found that *P. tricornutum* cells produce ovothiol B. By real-time PCR and HPLC quantitative analysis, we have demonstrated that the methodology of genetic engineering here described is successful in modulating *ovoA* expression and increasing ovothiol production. Most importantly, by confocal microscopy we have discovered that OvoA localizes in the mitochondria of this diatom, thus opening new avenues for the understanding of the role of these compounds in the cellular redox homoeostasis of microalgae.

## 2. Methods

### 2.1. Preparation of the overexpressing constructs

The expression constructs have been prepared using the Gibson assembly methodology and the NEBuilder Assembly Tool (http://nebuilder.neb.com/) following the manufacturer's instructions.

**Figure 2.**
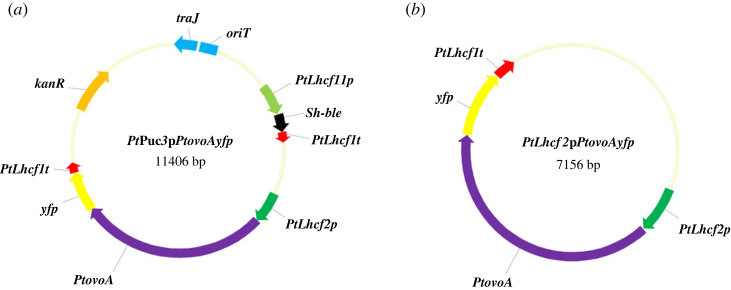
Overexpression constructs. (*a*) *Pt*Puc3p*PtovoAyfp*, the cargo vector for bacterial conjugation, included overexpression cassettes for antibiotic (*Sh*-ble) selection and for *P. tricornutum PtovoA* fused to *yfp* (Yellow Fluorescent Protein) along with resistance to kanamycin (*KanR*), transfer origin (*oriT*) sequence and *oriT* recognizing protein (*traJ*). (*b*) *PtLhcf2*p*PtovoAyfp*, the vector for biolistic transformation, included a cassette for the overexpression of *P. tricornutum PtovoA* fused to *yfp*. *PtLhcf11*p, *P. tricornutum Lhcf11* promoter; *PtLhcf1*t, *P. tricornutum Lhcf1* terminator; *PtLhcf2*p, *P. tricornutum Lhcf2* promoter; *PtovoA*, *P. tricornutum ovoA* coding sequence.

For *Pt*Puc3p*PtovoAyfp* (figure 2*a*), the following templates have been used: *Pt*Puc3p (https://www.addgene.org/62863/sequences/), containing the kanamycin and bleomycin (*Sh*-Ble) expressing cassettes, for the backbone vector, with the primer pair p*Pt*Puc3_fwd and p*Pt*Puc3_rev; *FcpBGus*At for the *Lhcf2* promoter with the primer pair *Lhcf2*p_fwd and *Lhcf2*p_rev; *PmH4*p*MRP3yfp* for the *yfp* coding sequence, with the primer pair *yfp-Lhcf1*t_fwd and *yfp-Lhcf1*t_rev; the *P. tricornutum* cDNA for the coding sequence of *PtovoA* (GenBank OP503499) without the stop codon, with the primer pair *ovo*_fwd and *ovo*_rev.

For *PtLhcf2*p*PtovoAyfp* (figure 2*b*), the plasmid *PtLhcf2*p*Ptlahyfp* has been digested with *Eco*RI to obtain the backbone vector containing the *Lhcf*2 promoter and the *yfp* coding sequence and the coding sequence of *PtovoA* without the stop codon has been amplified on *P. tricornutum* cDNA from wild-type strain with the primer pair *ovo*fw*Eco*RI and *ovo*dw*Eco*RI.

The assembled constructs were transformed in MAX Efficiency Stbl2 Competent Cells (Invitrogen) using kanamycin as selective antibiotic.

### 2.2. Cell cultures

The CCMP632 strain of *P. tricornutum* Bohlin was obtained from the Provasoli-Guillard National Center for Culture of Marine Phytoplankton. Cultures of axenic wild type and transformed strains were grown in F/2 -Si medium [42] at 18°C under white fluorescent lights (90 µmol photons m^−2^ s^−1^), in a 12 h light/12 h dark photoperiod.

### 2.3. *Phaeodactylum tricornutum* transformation by bacterial conjugation and biolistic transformation

The plasmid *Pt*Puc3p*PtovoAyfp* (cargo vector) was chemically transformed in *E. coli* DH10B cells containing the conjugation vector pTA-Mob (conjugation vector), bearing the gentamycin resistance [43]. *E. coli* DH10B, resistant to gentamycin and kanamycin, containing conjugation and cargo vectors were inoculated the day before the conjugation for overnight growth and used for the conjugation of *P. tricornutum* performed as in [44].

*PtLhcf2*p*PtovoAyfp* and *PtLhcf1*p*Sh*Ble (expressing the *Sh*-Ble gene conferring resistance to the phleomycin antibiotic) constructs have been introduced in *P. tricornutum* cells by means of microparticle bombardment using a Biolistic PDS-1000/He Particle Delivery System (Bio-Rad) as described in [45].

### 2.4. Selection of resistant clones

Resistant clones have been selected on plates prepared with F/2-Si medium diluted with 50% distilled water, 1% agar, and phleomycin (Invitrogen) (20 µg ml^−1^ and 50 µg ml^−1^, for conjugation and biolistic transformation, respectively).

Colony PCR was performed on cell lysates as in [11] with the primer pair *Lhcf2*pfw - *ovo1*rv to ascertain the presence of the episome (*Pt*Puc3p*ovoAyfp*) in the cells transformed by *E. coli* conjugation, or with *Lhcf2*pfw - *ovo1*rv and *ovo1*fw - *yfp*dw primer pairs (electronic supplementary material, table S1) to ascertain the presence of the transgene in the cells transformed with the biolistic method. The PCR products were analysed by electrophoresis on agarose gel (1% agarose w/v).

### 2.5. *PtovoA* subcellular localization

The OvoA protein sequence from *P. tricornutum* was scanned using the online tool for subcellular localization prediction WoLF PSORT link: https://wolfpsort.hgc.jp/. After conversion of protein amino acid sequences into numerical localization features, WoLF PSORT uses a simple k-nearest neighbour classifier for prediction. The dataset used to train WoLF PSORT contains over 12 000 animal sequences and more than 2000 plant and fungi sequences, respectively, and gives a summary line where the numbers indicate the number of nearest neighbours to the query which localize to each site [46]. Mitofates server (https://mitf.cbrc.pj.aist.go.jp/MitoFates/cgi-bin/top.cgi) [47] was employed for identifying the presence of any putative mitochondrial pre-sequences and cleavage sites within OvoA. For microscopic analysis a confocal laser scanning Leica TCS SP8 was used. Chlorophyll autofluorescence and YFP fluorescence were excited at 510 nm and detected at 650–740 nm and 540–560 nm, respectively. MitoTracker Orange (Molecular Probes) with excitation at 554 nm and emission at 576 nm, was used for mitochondria staining, following manufacturer's instructions.

### 2.6. Cell harvesting

Wild-type and transgenic cells were collected at different phases of the growth curve: T1 (day 3) corresponding to the early exponential phase, T2a and T2b (day 5) corresponding to the late exponential phase and T3 (day 7) corresponding to the beginning of the stationary phase. In particular, T1, T2a and T3 cells were collected 3 h after the onset of light, while T2b was collected 9 h after the onset of light. Cells were harvested by centrifugation at 2500×g for 15 min and frozen in liquid nitrogen.

### 2.7. RNA extraction and cDNA synthesis

Total RNA was extracted from wild type and transformed *P. tricornutum* cells as described in [48]. RNA concentration was determined using a Qubit 2.0 Fluorometer (Invitrogen) and qualitatively estimated by gel electrophoresis (1% agarose w/v). 200 ng of total extracted RNA was used to synthesize cDNA with PrimeScript Reverse Transcription Kit (TakaraBio) according to the manufacturer's instructions.

### 2.8. *PtovoA* expression analysis

1 µl of a 1 : 2 dilution of cDNA was used as template to amplify the *PtovoA* transcript using 0.4 µM final concentration of the primers *ovo1*fw and *ovo1*rv, using *RPS* (Ribosomal protein small subunit 30S; ID 10847) as reference gene [49]. Real-Time PCR amplification was performed using Power SYBR Green PCR Master Mix 2X (Applied Biosystem) in a final volume of 10 µl. Each reaction was tripled for both genes in each sample using 384- well plates (BioRad) in the ViiA 7 Real-Time PCR System (Thermo Fischer).

Data obtained were analysed with the ViiA 7 Real-Time PCR system software, and fold-changes were obtained with the Relative Expression Software Tool-Multiple Condition Solver (REST-MCS) [50].

### 2.9. Chemical identification and quantification of ovothiol

Thiols identification and quantitative determination was achieved by HPLC and UPLC-HR-ESI-MS analysis of the 4-bromomethyl-7-methoxycoumarin (BMC) derivatives. For the preparation of thiol BMC-derivatives, 10 mg of freeze dried cells were rehydrated with 20 µl of water, spiked with 10 µl of 1 mM *N*-Acetyl-cysteine (NAC) as internal standard and lysed by extensive vortexing with 90 µl of extraction buffer (AcCN : 0.75 M HClO_4_, 1 : 2). The samples were centrifuged (5 min, 16000×*g*) to remove insoluble cellular debris and 100 *μ*l of cleared lysate were neutralized by the addition of 15 µl of 2 M K_2_CO_3_. The mixture was centrifuged (2 min, 16000×*g*) to remove excess potassium perchlorate and 100 µl of supernatant were basified with 10 µl of Li_2_CO_3_ 50 mM. Cellular thiols were reduced with 3 µl of 200 mM DTT (5 min) before adding 25 µl of 20 mM BMC in DMSO (30 min, in the dark). The mixture was acidified by addition of 10 µl of 10% formic acid and vortexed extensively to remove CO_2_ and excess BMC prior to HPLC analysis. Initial LC-MS identification of ovothiol B was performed in the conditions described in [16] and achieved by identification of a HR-ESI-MS peak corresponding to its BMC-alkylated derivative (*m*/*z*_calc. = 404.1275, measured *m*/*z* = 404.1258, Δ = −4.2 ppm) and by coelution with an authentic sample produced in the laboratory of Prof. FP. Seebeck. Quantitation of the thiol-BMC conjugates was performed on an Agilent 1260 Infinity II system equipped with a Poroshell 120 EC-C18 column (4 µm, 150 × 4.6 mm, Agilent) and UV detection at 330 nm. The following gradient of solvent B (0.1% Formic Acid in AcCN) in A (0.1% Formic Acid) was used: 0.0–1.9 min, 2% B; 2.0 min, 3% B; 2.01–6.0 min, 9% B; 6.0–21.0 min, 9–45% B; 21.0–23.0 min, 45–90% B; 23.0–26.0 min, 90% B; 26.0–27.0 min, 90–2% B; 27.0–34.0, 2%. GSH concentrations were analysed as a control. Other thiols, available in the laboratory, like 5-thiohistidine, ovothiol A and ergothioneine, were used as standard for elution to assess also their absence/presence.

### 2.10. Statistical analyses

To evaluate gene expression and metabolite results, statistical analyses of data were performed by two-way ANOVA. Differences among strains and time course were evaluated by the Tukey's post hoc test (*p* < 0.05). All statistical analyses were performed with the statistical software package Prism 6 (GraphPad Software Inc.).

## 3. Results

### 3.1. Cloning of *PtovoA* and selection of transformant strains

**Figure 3.**
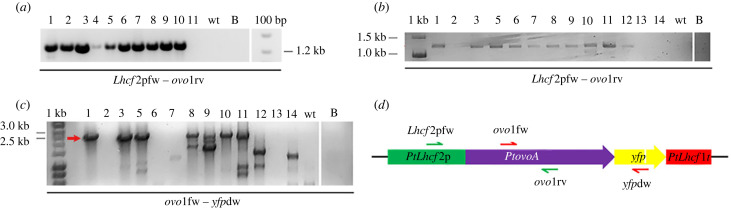
Electrophoresis of the resistant clones obtained by colony PCR for (*a*) bacterial conjugation (lanes 1–11), positive clones showed a band of 1205 bp, and (*b,c*) biolistic transformation (lanes 1–14). Positive clones showed the expected bands of (*b*) 1237 bp (lanes 1 and 3–12) and (*c*) 2619 bp (indicated by red arrow, lanes 1, 3, 5, 8, 10). Additional smaller bands in (*c*) indicate the possible presence of truncated versions of the construct. wt, wild type; B, blank (no template); 1 kb, molecular weight ladder; 100 bp, molecular weight ladder. (*d*) Outline of the *PtovoA-yfp* expression cassette with the indication of the primers used for the colony PCR.

We transformed *P. tricornutum* cells with a plasmid encoding the *PtovoA* sequence in frame with the fluorescent protein YFP using both bacterial conjugation and the biolistic method (figure 2*a,b*) in order to overexpress the enzyme and follow its subcellular localization. Ten out of 11 resistant clones obtained through *E. coli* conjugation showed the expected PCR amplicon of 1205 bp (figure 3*a*) corresponding to a fragment encompassing the *P. tricornutum Lhcf*2 promoter and the *PtovoA* coding sequence (figure 3*d*), while five out of 13 resistant clones obtained through biolistic transformation showed the two expected PCR amplicons of 1237 bp (figure 3*b*) and 2619 bp (figure 3*c*), corresponding to two fragments encompassing the *P. tricornutum Lhcf*2 promoter and the *PtovoA* coding sequence (figure 3*d*) and the *PtovoA* and the *yfp* coding sequences, respectively (figure 3*d*). The three amplicons were not detected in the WT strain (figure 3*a–c*).

### 3.2. *PtovoA* cellular localization

To predict the subcellular localization site of OvoA in diatoms*,* we used the WoLF PSORT software based on the amino acid sequence of *Pt*OvoA. *Pt*OvoA was predicted to localize with the highest probability in the mitochondrion (table 1). Predictions of MitoFates server [47] revealed that OvoA protein has a mitochondrial targeting pre-sequence and cleavage sites for mitochondrial processing peptidases (MPP) and intermediate cleavage peptidase55 (ICP55) predicted to cleave respectively the pre-sequence at serine and leucine residues (28 and 29 amino acid position) (table 1). OvoA protein is also predicted to have a translocase of the outer membrane (TOM20) recognition motif sequence positioned from 17 to 21 amino acid residues and an amphipathic alpha-helix located from 13 to 22 amino acid residues.

**Table 1.** Predicted subcellular localization of the protein obtained using WoLF PSORT. The numbers indicate the number of nearest neighbours to the query which localize to each site, adjusted to account for the possibility of dual localization. The prediction is carried out for three different datasets (animal, plant and fungi). Predicted mitochondrial targeting presequence with cleavage site for MPP (S28) and Icp55 (L29) obtained using Mitofates. The two amino acid residues are indicated in bold in the protein sequence. The TOM20 recognition motif is boxed; the amphipathic alpha-helix motif is underlined.

animal	mitochondria: 25, plasma membrane: 2.5, membrane and cytoskeleton: 2, extracellular: 1, nuclear: 1, peroxisome: 1, lysosome: 1
plant	mitochondria: 13, chloroplast: 1
fungi	mitochondria: 18, extracellular: 5, cytoplasm: 2, peroxisome: 2
probability of presequence	0.984
prediction	possessing mitochondrial presequence
cleavage site	S28(MPP), L29(Icp55)
net charge	0.179
TOM20 recognition motif	17–21 aa
amphypathic alpha-helix	13–22 aa
N-terminal amino acids and motifs: MMLSPQAARMGTVCTTLRKIVSTARQRSLSHSAFASVRSETAFGARAFSTRSTLAALEDSDEDLTFRSQGHAAAAAA	

**Figure 4.**
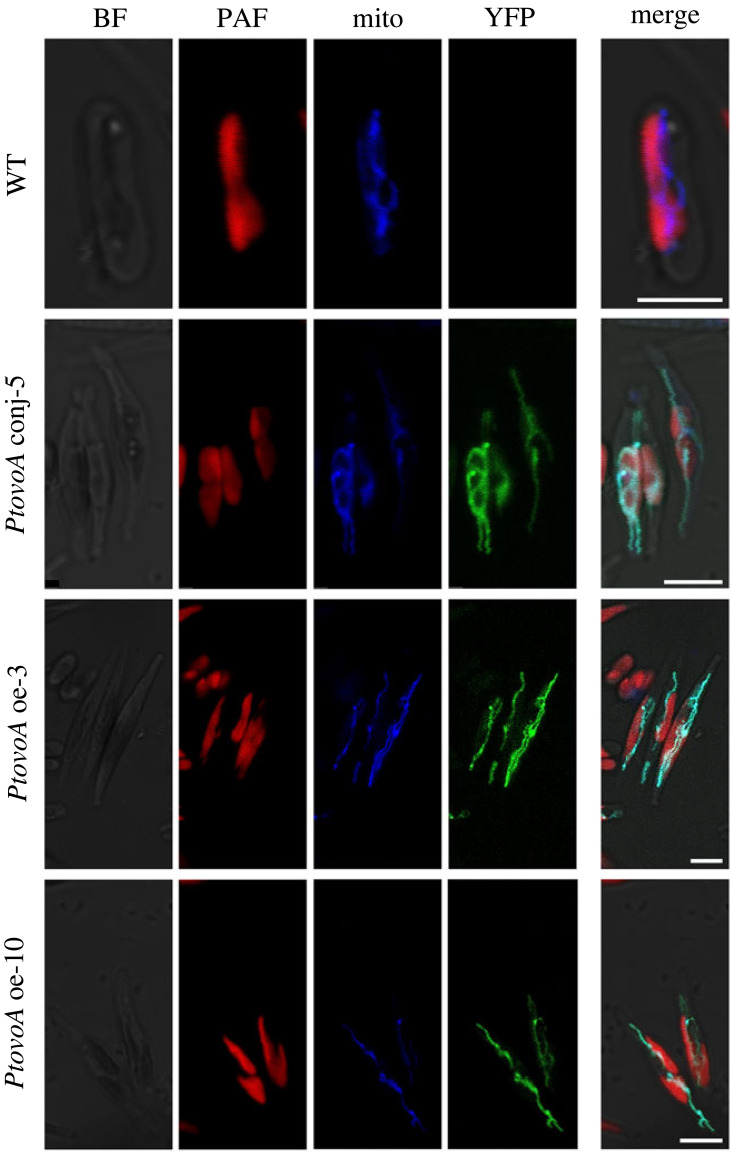
Confocal laser scanning microscopy images of *P. tricornutum* wild type (WT) and transformant cells (*PtovoA*conj-5, *PtovoA* oe-3 and *PtovoA* oe-10) in bright field (BF), showing a red signal corresponding to the autofluorescing chlorophyll (PAF) and a green signal corresponding to the fusion protein *PtovoA*-*yfp*. The blue signal corresponds to the MitoTracker staining in the mitochondrion (Mito). Panels on the right show a merge of the different signals (Merge). Scale bars: 5 µm.

The cellular localization prediction of *Pt*OvoA was confirmed by visualization with confocal laser scanning microscopy of the overexpressed YFP-tagged proteins. Regardless of the method used, in three overexpressing clones, one derived from bacterial conjunction (here named *PtovoA* conj-5), and two obtained by the biolistic method (here named *PtovoA* oe-3 and *PtovoA* oe-10), the YFP signal was visualized in the mitochondria along with the co-localizing MitoTracker signal (figure 4).

### 3.3. Evaluation of *PtovoA* gene overexpression during *P. tricornutum* growth

**Figure 5.**
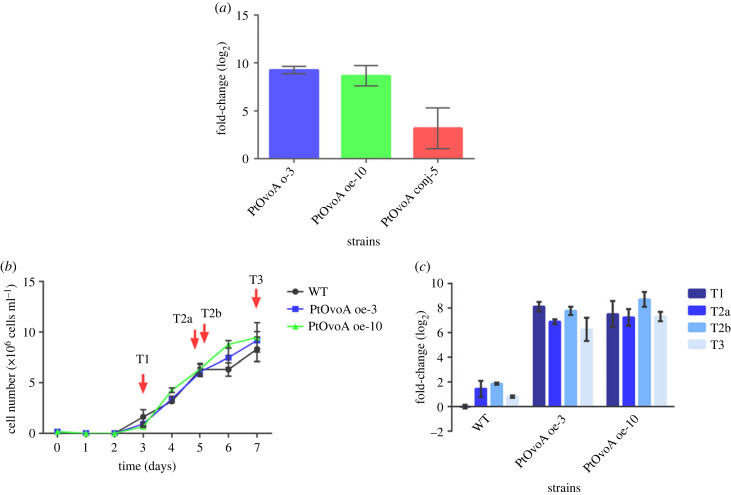
Growth curve and *PtovoA* gene expression levels in *P. tricornutum* WT and overexpressing strains. (*a*) *PtovoA* relative gene expression levels in *PtovoA* oe-3, *PtovoA* oe-10 and *PtovoA* conj-5 strains compared to WT set as baseline. Error bars represent the standard deviation (*n* = 3 technical replicates). (*b*) Growth curves for *P. tricornutum* WT, *PtovoA* oe-3 and *PtovoA* oe-10 strains. Each point represents the mean of three replicates ± s.d. (*n* = 3 independent experiments). Red arrows indicate the time of sampling for gene expression and biochemical analyses. (*c*) *PtovoA* relative gene expression levels in *PtovoA* oe-3 and *PtovoA* oe-10 strains at different time points compared to WT at T1 set as zero. Error bars represent the standard deviation (*n* = 3 technical replicates). Significant differences in relative gene expression can be considered when the fold-change is greater than 2.

The overexpression of the *PtovoA* gene was evaluated in the representative clones obtained through the two alternative transformation approaches. Overexpression levels in *PtovoA* oe-3, *PtovoA* oe-10 and *PtovoA* conj-5 resulted in 9.26 ± 0.39, 8.65 ± 1.06 and 3.19 ± 2.13 fold changes, respectively, compared to the WT, considered as baseline (figure 5*a*). These data indicated that the biolistic method led to a significantly higher overexpression of the gene compared to the *E. coli* conjugation. Therefore, we decided to use the two biolistic clones for further analyses on metabolite overproduction.

Cells of the two strains *PtovoA* oe-3, *PtovoA* oe-10 compared to WT were harvested at different growth phases, from early and late exponential phase to the beginning of the stationary phase (T1, T2 and T3) and two diel time points (T2a and T2b), to evaluate *PtovoA* gene overexpression levels and the optimal harvesting time point to obtain the maximum yield of the metabolite. The growth properties of *PtovoA* oe-3 and *PtovoA* oe-10 strains were not different from those of the WT (figure 5*b*). *PtovoA* gene expression levels in WT, *PtovoA* oe-3 and *PtovoA* oe-10 strains compared to the housekeeping gene *RPS,* showed a significant increase, with a 6.2 to 8.7 folds change of the *PtovoA* transcript in the transgenic strains compared to WT. Although the expression of *ovoA* in the transgenic strains is driven by the light-dependent *Lhcf2* promoter, the levels of expression in the transgenic strains seemed independent from the growth phase and the diel time, similarly to the endogenous expression of the *ovoA* gene (FC < 2, figure 5*c*).

### 3.4. Thiols analysis and identification of ovothiol B in *P. tricornutum* cells

**Figure 6.**
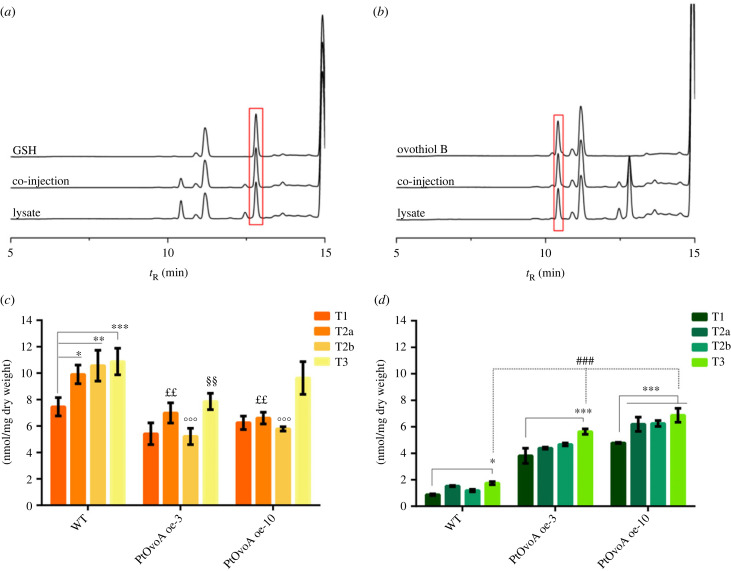
HPLC analysis of cellular thiols in *P. tricornutum* cells. (*a*) HPLC traces of authentic samples of GSH, and co-injection with the lysate of *P. tricornutum* cells. The red box highlights the peaks corresponding to GSH. (*b*) HPLC traces of authentic samples of ovothiol B and co-injection with *P. tricornutum* lysate. The red box highlights the peaks corresponding to ovothiol B. (*c*) GSH and (*d*) Ovothiol B molar content per mg dry weight in WT, *PtovoA* oe-3 and *Ptovoa* oe-10 over the growth curve. Data were analysed by Two-way ANOVA followed by Tukey post-test. Bars represent mean ± s.d. *p < 0.05; **p < 0.01; ***p < 0.001. ### indicates values that are significantly higher than ovothiol B nmol mg^−1^ dry weight in WT T3 (*p*-value < 0.001); ££, °°°, §§ indicates values significantly lower than GSH nmol mg^−1^ dry weight in the corresponding time points of WT (*p* < 0.01 for ££ and §§; *p* < 0.001 for °°°).

To verify that both WT *P. tricornutum* and the overexpressing clones produced ovothiol, we evaluated the thiol content of cells collected at T2, in the late exponential phase. To better compare the pools of the cellular thiols and highlight a possible mutual regulation, we confronted their molar abundance. Extracted compounds were alkylated with the thiol-specific electrophile BMC and analysed by reversed phase HPLC and UPLC-HR-ESI-MS. In WT cells, GSH, the main thiol, was present at a concentration of 9.8 ± 0.2 nmol mg^−1^ dry weight. Interestingly, by HR-ESI-MS and by co-elution with an authentic sample (figure 6*a,b*), we could identify in the extract the signal for the BMC-alkylated derivative of ovothiol B (*m*/*z*_calc. = 404.1275, measured *m*/*z* = 404.1258, Δ < 5ppm). No traces of known alternative thiols, such as ergothioneine, 5-thiohistidine and ovothiol A, were detected. The quantitative analysis showed that the amount of ovothiol B in *Pt*WT was 1.44 nmol mg^−1^ dry weight. Preliminary quantitative analysis of the content of ovothiol B in the bacterial conjugated transformants pointed to a two-fold increase in the levels of the metabolite compared to *Pt*WT. By comparison, the selected biolistic transgenic lines produced up to 4-fold the amount of ovothiol B (*PtovoA* oe-3: 3.7 ± 0.1, *PtovoA* oe-10: 5.6 ± 0.1 nmol mg^−1^ dry weight) compared to *Pt*WT, while exhibiting slightly lower concentrations of GSH (6.8 ± 0.2 nmol mg^−1^ dry weight).

We then evaluated the production of ovothiol B in the biolistic clones during the growth of *P. tricornutum* at the same time points considered for the gene expression analysis (figure 6*c,d*)*.* To better understand if the overproduction of this metabolite could have consequences on the pools of other cellular antioxidant, we compared its molar abundance with that of GSH, physiologically predominant. The data showed that ovothiol B accumulates throughout the growth, reaching its highest levels in the stationary phase (T3), with a peak value of 6.5 ± 0.1 nmol mg^−1^ dry weight in *PtovoA* oe-10. The level of GSH, which remains the main cellular thiol, follows the same trend, but while in *Pt*WT the GSH content is 10.7 ± 0.3 nmol mg^−1^ dry weight at T3, the levels of GSH in the transformant clones decrease slightly in response to the accumulation of ovothiol B, suggesting a mutual regulation of the two redox species (figure 6*c,d*).

## 4. Discussion

In this paper, we have reported for the first time the occurrence of ovothiol B, previously found in the centric diatom *S. marinoi* [16], also in the evolutionary younger pennate diatom *P. tricornutum*. This finding suggests a conserved metabolic fingerprint for the same form of ovothiol in diatoms. We have found that WT *P. tricornutum* cells in physiological conditions produce significantly higher amounts of ovothiol B (0.31 ± 0.01 µg mg^−1^ dry weight) compared to *S. marinoi* (approximately 0.04 µg mg^−1^ dry material [16], thus confirming that this diatom can be considered a more suitable system for ovothiol production.

In this work, we have used two different methodologies to overexpress *PtovoA* in *P. tricornutum*, the bacterial conjugation and the biolistic method. In the bacterial conjugation, the presence of a single-copy non-integrated episome containing the transgene does not alter the genome sequence, avoiding functional alterations to the cells, and therefore it represents a more powerful and effective method for functional studies [44]. On the other hand, the biolistic transformation allows the integration of the transgene in the genome and offers the technical advantage of yielding stable clones that do not require continuous antibiotic selection in the medium. Moreover, the opportunity to have multiple transgene insertions permits to isolate clones with higher levels of expression and hence to obtain a higher amount of the desired metabolite [8,45].

By fusing *PtovoA* gene with *yfp*, we observed for the first time that OvoA protein localizes in the mitochondrion. Therefore, we can assume at least in diatoms, that after the biosynthesis in the endoplasmic reticulum the protein can translocate to mitochondria, as also suggested by the existence of an N-terminal mitochondrial targeting sequence. This represents a very exciting functional finding since, so far, no subcellular localization of OvoA had been described neither in invertebrates or microalgae. However, our bioinformatics analysis predicts a target sequence in OvoA for mitochondrion also in animals, thus extending our observations also to the other kingdoms. Indeed, protein localization in the subcellular compartment may offer insightful suggestions on the OvoA biological function, which is far from being completely understood, particularly in diatoms, where the metabolite was only recently identified [16]. Thanks to their ability to scavenge peroxides and participate to redox exchange with GSH, ovothiols have been proposed to protect eggs and early embryos against the oxidative burst occurring at fertilization and during development in sea urchins [38,51], to be involved in the maturation and differentiation of sea urchins female gonads [52], to protect against the oxidative stress produced by the immune response of the host in Trypanosoma species [53], and against environmental pollutants in sea urchins and *Mytilus galloprovincialis* [26,54]. Therefore, the mitochondrial localization of the OvoA enzyme in diatoms represents a very interesting finding in light of the unique redox properties of ovothiol. Indeed, mitochondria represent the energy centre of the cell, in which the final reaction of cellular respiration, i.e. the reduction of oxygen to water, takes place, producing reactive oxygen species (ROS) as by-products. Therefore, the role of a potent antioxidant as ovothiol may be crucial to maintain mitochondrial redox homeostasis. Interestingly, we found that the accumulation of ovothiol in overexpressing diatoms was accompanied by a decrease in GSH content, further suggesting the role of ovothiol as an alternative antioxidant to GSH. In almost all organisms, from bacteria to mammals, GSH is the major antioxidant cellular thiol in the cell, being present from 1 to 10 mM concentrations. However, different alternative thiols have been identified in some microorganisms, such as bacillothiols in bacilli, trypanothione in Trypanosoma species, ergothionine in fungi and bacteria, which may be representative of evolutionary adaptation to different ecological niches [53]. Moreover, among the pool of antioxidants that protect mitochondria from ROS, GSH is thought to be essential for the organelle antioxidant function. Even if mitochondria cannot synthesize GSH *de novo*, its efficient transport from the cytosol is sufficient to maintain the mitochondrial redox status [55]. In this case, the proteins involved in GSH metabolism are not localized in the mitochondria, but the tripeptide is imported through a dedicated membrane transporter [56]. In the case of *P. tricornutum*, the localization of OvoA in mitochondria suggests that the biosynthesis of ovothiol actually occurs in mitochondria, thus indicating a more specific function. This finding is particularly excited if we consider that in diatoms mitochondrial respiration contributes to the optimization of photosynthesis [57].

In addition to functional studies, our experiments proved that the expression levels of *PtovoA* and the accumulation of ovothiol in conjugated mutants were much lower compared to the biolistic ones. Therefore, we decided to focus on the latter for perspective biotechnological applications.

Indeed, the use of *P. tricornutum* cultures offers many advantages over other organisms, due to their high productivity, easy scale-up of upstream and downstream processing, high stability to seasonal changes, easy and fast biomass production and the possibility to be grown in outdoor reactors for the production of different compounds with biotechnological application [58]. For these reasons, we also explored the possibility to use *P. tricornutum* as a cell factory to overexpress the *ovoA* gene in order to develop an eco-sustainable protocol for ovothiol production.

Our results showed that in WT diatoms the endogenous expression levels of *PtovoA* was not significantly affected by the growth phase and the diel time. The same was true for the transgenic biolistic strains, where overexpression is driven by the strong, light-dependent *Lhcf2* promoter, and where higher expression levels of *PtovoA* are stable throughout the growth curve. On the other hand, the final amount of ovothiol B reaches the highest levels in the stationary phase, most likely as a result of accumulation over time, slowly approaching a plateau as the growth proceeds.

The accumulation of the final metabolite that we observed in the most productive strains was roughly 5-fold higher than the basal production levels in WT *P. tricornutum* (1.4 µg mg^−1^ versus 0.3 µg mg^−1^ dry weight), approaching the gold standard for the levels of ovothiol in sea urchin eggs, approximately 4 µg mg^−1^ dried cells [30]. However, diatoms can be grown in bioreactors to obtain greater amounts of the metabolite, while sea urchin eggs do not represent a sustainable source. At the moment, we cannot rule out that higher amounts of ovothiol B could be produced in the *P. tricornutum* system by further manipulation of the ovothiol biosynthetic pathway, for instance through the concomitant overexpression of the OvoB enzyme, or by the fine adjustment of the culture conditions. At the same time, the observed levels of metabolite might be approaching an upper threshold, before becoming incompatible with the correct redox homeostasis of the cell. Further studies will clarify these points.

Considering that bacterial OvoA is a promiscuous enzyme which has been shown to accept different methylated form of histidine and produce different forms of ovothiol [59], it is likely that *Pt*OvoA in physiological conditions accepts a mono-methyl histidine to give ovothiol B. Therefore, the possibility to use our diatoms-based engineered system to produce also ovothiol A, providing for example an excess of histidine to the cell cultures, represents an excited hypothesis to test in future experiments of synthetic biology.

## 5. Conclusion

The genetic engineered system here described has allowed us to discover the mitochondrial localization of OvoA in diatoms, giving insightful suggestions on its biological function. In addition, this system offers several competitive advantages, because it allows production of natural π-methylated ovothiols in an eco-sustainable way, without toxic by-products and expensive procedures. The production of ovothiols may be relevant for the pharmaceutical, nutraceutical and cosmeceutical sectors, due to the increasing evidence of their therapeutic value.

## Data Availability

The coding sequence of *P. tricornutum ovoA* has been submitted on GenBank and is publicly available with the accession number OP503499. The data are provided in electronic supplementary material [60].

## References

[1] Vancaester E, Depuydt T, Osuna-Cruz CM, Vandepoele K. 2020 Comprehensive and functional analysis of horizontal gene transfer events in diatoms. Mol. Biol. Evol. **37**, 3243-3257. (10.1093/molbev/msaa182) 32918458

[2] Medlin L. 2016 Evolution of the diatoms: major steps in their evolution and a review of the supporting molecular and morphological evidence. Phycologia **55**, 79-103. (10.2216/15-105.1)

[3] Bowler C et al. 2008 The Phaeodactylum genome reveals the evolutionary history of diatom genomes. Nature **456**, 239-244. (10.1038/nature07410) 18923393

[4] Kroth PG et al. 2018 Genome editing in diatoms: achievements and goals. Plant Cell Rep. **37**, 1401-1408. (10.1007/s00299-018-2334-1) 30167805

[5] Sharma N, Simon DP, Diaz-Garza AM, Fantino E, Messaabi A, Meddeb-Mouelhi F, Germain H, Desgagné-Penix I. 2021 Diatoms biotechnology: various industrial applications for a greener tomorrow. Front. Mar. Sci. **8**, 636613. (10.3389/fmars.2021.636613)

[6] Santin A, Russo MT, Ferrante MI, Balzano S, Orefice I, Sardo A. 2021 Highly valuable polyunsaturated fatty acids from microalgae: strategies to improve their yields and their potential exploitation in aquaculture. Molecules **26**, 7697. (10.3390/molecules26247697) 34946780 PMC8707597

[7] Santin A, Balzano S, Russo MT, Palma Esposito F, Ferrante MI, Blasio M, Cavalletti E, Sardo A. 2022 Microalgae-based PUFAs for food and feed: current applications, future possibilities, and constraints. J. Mar. Sci. Eng. **10**, 844. (10.3390/jmse10070844)

[8] George J, Kahlke T, Abbriano RM, Kuzhiumparambil U, Ralph PJ, Fabris M. 2020 Metabolic engineering strategies in diatoms reveal unique phenotypes and genetic configurations with implications for algal genetics and synthetic biology. Front. Bioeng. Biotechnol. **8**, 513. (10.3389/fbioe.2020.00513) 32582656 PMC7290003

[9] Butler T, Kapoore RV, Vaidyanathan S. 2020 *Phaeodactylum tricornutum*: a diatom cell factory. Trends Biotechnol. **38**, 606-622. (10.1016/j.tibtech.2019.12.023) 31980300

[10] Levitan O, Dinamarca J, Hochman G, Falkowski PG. 2014 Diatoms: a fossil fuel of the future. Trends Biotechnol. **32**, 117-124. (10.1016/j.tibtech.2014.01.004) 24529448

[11] Daboussi F et al. 2014 Genome engineering empowers the diatom *Phaeodactylum tricornutum* for biotechnology. Nat. Commun. **5**, 3831. (10.1038/ncomms4831) 24871200

[12] Xue J, Niu Y-F, Huang T, Yang W-D, Liu J-S, Li H-Y. 2015 Genetic improvement of the microalga *Phaeodactylum tricornutum* for boosting neutral lipid accumulation. Metab. Eng. **27**, 1-9. (10.1016/j.ymben.2014.10.002) 25447640

[13] Zulu NN, Zienkiewicz K, Vollheyde K, Feussner I. 2018 Current trends to comprehend lipid metabolism in diatoms. Prog. Lipid Res. **70**, 1-16. (10.1016/j.plipres.2018.03.001) 29524459

[14] Hao X et al. 2018 Enhanced triacylglycerol production in the diatom *Phaeodactylum tricornutum* by inactivation of a Hotdog-fold thioesterase gene using TALEN-based targeted mutagenesis. Biotechnol. Biofuels **11**, 312. (10.1186/s13068-018-1309-3) 30455741 PMC6231261

[15] O'Neill EC, Trick M, Hill L, Rejzek M, Dusi RG, Hamilton CJ, Zimba PV, Henrissat B, Field RA. 2015 The transcriptome of *Euglena gracilis* reveals unexpected metabolic capabilities for carbohydrate and natural product biochemistry. Mol. Biosyst. **11**, 2808-2820. (10.1039/C5MB00319A) 26289754

[16] Milito A, Castellano I, Burn R, Seebeck FP, Brunet C, Palumbo A. 2020 First evidence of ovothiol biosynthesis in marine diatoms. Free Radic. Biol. Med. **152**, 680-688. (10.1016/j.freeradbiomed.2020.01.010) 31935446

[17] Holler TP, Hopkins PB. 1988 Ovothiols as biological antioxidants. The thiol groups of ovothiol and glutathione are chemically distinct. J. Am. Chem. Soc. **110**, 4837-4838. (10.1021/ja00222a057)

[18] Zoete V, Bailly F, Vezin H, Teissier E, Duriez P, Fruchart JC, Catteau JP, Bernier JL. 2000 4-Mercaptoimidazoles derived from the naturally occurring antioxidant ovothiols 1. Antioxidant properties. Free Radic. Res. **32**, 515-524. (10.1080/10715760000300521) 10798717

[19] Seebeck FP. 2013 Thiohistidine biosynthesis. CHIMIA **67**, 333-333. (10.2533/chimia.2013.333) 23863267

[20] Palumbo A, Castellano I, Napolitano A. 2018 Ovothiol: a potent natural antioxidant from marine organisms. In Blue biotechnology (eds S La Barre, SS Bates), pp. 583-610. Chichester, UK: John Wiley & Sons.

[21] Castellano I, Seebeck FP. 2018 On ovothiol biosynthesis and biological roles: from life in the ocean to therapeutic potential. Nat. Prod. Rep. **35**, 1241-1250. (10.1039/C8NP00045J) 30052250

[22] Milito A, Orefice I, Smerilli A, Castellano I, Napolitano A, Brunet C, Palumbo A. 2020 Insights into the light response of *Skeletonema marinoi*: involvement of ovothiol. Mar. Drugs **18**, 477. (10.3390/md18090477) 32962291 PMC7551349

[23] Weaver KH, Rabenstein DL. 1995 Thiol/disulfide exchange reactions of ovothiol A with glutathione. J. Org. Chem. **60**, 1904-1907. (10.1021/jo00111a065)

[24] Osik NA, Zelentsova EA, Tsentalovich YP. 2021 Kinetic studies of antioxidant properties of ovothiol A. Antioxidants **10**, 1470. (10.3390/antiox10091470) 34573105 PMC8470380

[25] Palumbo A, Misuraca G, d'Ischia M, Donaudy F, Prota G. 1984 Isolation and distribution of 1-methyl-5-thiol-l-histidine disulphide and a related metabolite in eggs from echinoderms. Comp. Biochem. Physiol. Part B Comp. Biochem. **78**, 81-83. (10.1016/0305-0491(84)90149-4)

[26] Castellano I, Migliaccio O, D'Aniello S, Merlino A, Napolitano A, Palumbo A. 2016 Shedding light on ovothiol biosynthesis in marine metazoans. Sci. Rep. **6**, 21506. (10.1038/srep21506) 26916575 PMC4768315

[27] Turner E, Klevit R, Hager LJ, Shapiro BM. 1987 Ovothiols, a family of redox-active mercaptohistidine compounds from marine invertebrate eggs. Biochemistry **26**, 4028-4036. (10.1021/bi00387a043) 3651433

[28] Brancaccio M, Tangherlini M, Danovaro R, Castellano I. 2021 Metabolic adaptations to marine environments: molecular diversity and evolution of ovothiol biosynthesis in bacteria. Genome Biol. Evol. **13**, evab169. (10.1093/gbe/evab169) 34272861 PMC8433421

[29] Gerdol M, Sollitto M, Pallavicini A, Castellano I. 2019 The complex evolutionary history of sulfoxide synthase in ovothiol biosynthesis. Proc. R. Soc. B **286**, 20191812. (10.1098/rspb.2019.1812) PMC6939259 31771466

[30] Russo GL, Russo M, Castellano I, Napolitano A, Palumbo A. 2014 Ovothiol isolated from sea urchin oocytes induces autophagy in the Hep-G2 cell line. Mar. Drugs **12**, 4069-4085. (10.3390/md12074069) 25003791 PMC4113815

[31] Brancaccio M, Russo M, Masullo M, Palumbo A, Russo GL, Castellano I. 2019 Sulfur-containing histidine compounds inhibit γ-glutamyl transpeptidase activity in human cancer cells. J. Biol. Chem. **294**, 14 603-14 614. (10.1074/jbc.RA119.009304) PMC6779435 31375562

[32] Brancaccio M, D'Argenio G, Lembo V, Palumbo A, Castellano I. 2018 Antifibrotic effect of marine ovothiol in an *in vivo* model of liver fibrosis. Oxid. Med. Cell. Longev. **2018**, 1-10. (10.1155/2018/5045734) PMC6311726 30647809

[33] Castellano I, Di Tomo P, Di Pietro N, Mandatori D, Pipino C, Formoso G, Napolitano A, Palumbo A, Pandolfi A. 2018 Anti-inflammatory activity of marine ovothiol A in an *in vitro* model of endothelial dysfunction induced by hyperglycemia. Oxid. Med. Cell. Longev. **2018**, e2087373. (10.1155/2018/2087373) PMC5932987 29849868

[34] Brancaccio M, Milito A, Viegas CA, Palumbo A, Simes DC, Castellano I. 2022 First evidence of dermo-protective activity of marine sulfur-containing histidine compounds. Free Radic. Biol. Med. **192**, 224-234. (10.1016/j.freeradbiomed.2022.09.017) 36174879

[35] Vamecq J, Maurois P, Bac P, Bailly F, Bernier J-L, Stables JP, Husson I, Gressens P. 2003 Potent mammalian cerebroprotection and neuronal cell death inhibition are afforded by a synthetic antioxidant analogue of marine invertebrate cell protectant ovothiols. Eur. J. Neurosci. **18**, 1110-1120. (10.1046/j.1460-9568.2003.02846.x) 12956711

[36] Braunshausen A, Seebeck FP. 2011 Identification and characterization of the first ovothiol biosynthetic enzyme. J. Am. Chem. Soc. **133**, 1757-1759. (10.1021/ja109378e) 21247153

[37] Naowarojna N et al. 2018 In vitro reconstitution of the remaining steps in ovothiol A biosynthesis: C–S lyase and methyltransferase reactions. Org. Lett. **20**, 5427-5430. (10.1021/acs.orglett.8b02332) 30141637

[38] Milito A, Cocurullo M, Columbro A, Nonnis S, Tedeschi G, Castellano I, Arnone MI, Palumbo A. 2022 Ovothiol ensures the correct developmental programme of the sea urchin *Paracentrotus lividus* embryo. Open Biol. **12**, 210262. (10.1098/rsob.210262) 35042403 PMC8767189

[39] Daunay S, Lebel R, Farescour L, Yadan J-C, Erdelmeier I. 2016 Short protecting-group-free synthesis of 5-acetylsulfanyl-histidines in water: novel precursors of 5-sulfanyl-histidine and its analogues. Org. Biomol. Chem. **14**, 10 473-10 480. (10.1039/C6OB01870J) 27759126

[40] Erdelmeier I, Daunay S. 2018 5-acylsulfanyl-histidine compounds as precursors of the corresponding 5-sulfanylhistidines and their disulfides. US9926300B2.

[41] Erdelmeier I, Daunay S. 2020 Novel 5-acylsulfanyl-histidine compounds as precursors of the corresponding 5-sulfanylhistidines and their disulfides. US20200017479A1.

[42] Guillard RRL. 1975 Culture of Phytoplankton for Feeding Marine Invertebrates. In Culture of marine invertebrate animals (eds WL Smith, MH Chanley), pp. 29-60. Boston, MA: Springer.

[43] Strand TA, Lale R, Degnes KF, Lando M, Valla S. 2014 A new and improved host-independent plasmid system for RK2-based conjugal transfer. PLoS ONE **9**, e90372. (10.1371/journal.pone.0090372) 24595202 PMC3940858

[44] Karas BJ et al. 2015 Designer diatom episomes delivered by bacterial conjugation. Nat. Commun. **6**, 6925. (10.1038/ncomms7925) 25897682 PMC4411287

[45] Falciatore A, Casotti R, Leblanc C, Abrescia C, Bowler C. 1999 Transformation of nonselectable reporter genes in marine diatoms. Mar. Biotechnol. N. Y. N **1**, 239-251. (10.1007/PL00011773) 10383998

[46] Horton P, Park K-J, Obayashi T, Fujita N, Harada H, Adams-Collier CJ, Nakai K. 2007 WoLF PSORT: protein localization predictor. Nucleic Acids Res. **35**, W585-W587. (10.1093/nar/gkm259) 17517783 PMC1933216

[47] Fukasawa Y, Tsuji J, Fu S-C, Tomii K, Horton P, Imai K. 2015 MitoFates: improved prediction of mitochondrial targeting sequences and their cleavage sites. Mol. Cell. Proteomics MCP **14**, 1113-1126. (10.1074/mcp.M114.043083) 25670805 PMC4390256

[48] Russo MT, Annunziata R, Sanges R, Ferrante MI, Falciatore A. 2015 The upstream regulatory sequence of the light harvesting complex Lhcf2 gene of the marine diatom *Phaeodactylum tricornutum* enhances transcription in an orientation- and distance-independent fashion. Mar. Genom. **24**, 69-79. (10.1016/j.margen.2015.06.010) 26117181

[49] Siaut M, Heijde M, Mangogna M, Montsant A, Coesel S, Allen A, Manfredonia A, Falciatore A, Bowler C. 2007 Molecular toolbox for studying diatom biology in *Phaeodactylum tricornutum*. Gene **406**, 23-35. (10.1016/j.gene.2007.05.022) 17658702

[50] Pfaffl MW, Horgan GW, Dempfle L. 2002 Relative expression software tool (REST) for group-wise comparison and statistical analysis of relative expression results in real-time PCR. Nucleic Acids Res. **30**, e36. (10.1093/nar/30.9.e36) 11972351 PMC113859

[51] Shapiro BM. 1991 The control of oxidant stress at fertilization. Science **252**, 533-536. (10.1126/science.1850548) 1850548

[52] Murano C, Zuccarotto A, Leone S, Sollitto M, Gerdol M, Castellano I, Palumbo A. 2022 A survey on the distribution of ovothiol and ovoA gene expression in different tissues and cells: a comparative analysis in sea urchins and mussels. Mar. Drugs **20**, 268. (10.3390/md20040268) 35447941 PMC9029387

[53] Ariyanayagam MF. 2001 Ovothiol and trypanothione as antioxidants in trypanosomatids. Mol. Biochem. Parasitol. **115**, 189-198. (10.1016/S0166-6851(01)00285-7) 11420105

[54] Diaz de Cerio O, Reina L, Squatrito V, Etxebarria N, Gonzalez-Gaya B, Cancio I. 2020 Gametogenesis-related fluctuations in ovothiol levels in the mantle of mussels from different estuaries: fighting oxidative stress for spawning in polluted waters. Biomolecules **10**, 373. (10.3390/biom10030373) 32121166 PMC7175103

[55] Quintana-Cabrera R, Bolaños JP. 2013 Glutathione and γ-glutamylcysteine in the antioxidant and survival functions of mitochondria. Biochem. Soc. Trans. **41**, 106-110. (10.1042/BST20120252) 23356267

[56] Wang Y et al. 2021 SLC25A39 is necessary for mitochondrial glutathione import in mammalian cells. Nature **599**, 136-140. (10.1038/s41586-021-04025-w) 34707288 PMC10981497

[57] Bailleul B et al. 2015 Energetic coupling between plastids and mitochondria drives CO2 assimilation in diatoms. Nature **524**, 366-369. (10.1038/nature14599) 26168400

[58] Rodolfi L, Biondi N, Guccione A, Bassi N, D'Ottavio M, Arganaraz G, Tredici MR. 2017 Oil and eicosapentaenoic acid production by the diatom Phaeodactylum tricornutum cultivated outdoors in Green Wall Panel (GWP®) reactors. Biotechnol. Bioeng. **114**, 2204-2210. (10.1002/bit.26353) 28627710 PMC5599966

[59] Song H, Leninger M, Lee N, Liu P. 2013 Regioselectivity of the oxidative C–S bond formation in ergothioneine and ovothiol biosyntheses. Org. Lett. **15**, 4854-4857. (10.1021/ol402275t) 24016264 PMC4166525

[60] Russo MT, Santin A, Zuccarotto A, Leone S, Palumbo A, Ferrante MI, Castellano I. 2023 The first genetic engineered system for ovothiol biosynthesis in diatoms reveals a mitochondrial localization for the sulfoxide synthase OvoA. Figshare. (10.6084/m9.figshare.c.6385934) PMC9890322 36722300

